# Accuracy and influencing factors of the Field Triage Decision Scheme for adult trauma patients at a level-1 trauma center in Korea

**DOI:** 10.1186/s12873-022-00637-1

**Published:** 2022-06-07

**Authors:** Byung Hee Kang, Kyoungwon Jung, Sora Kim, So Hyun Youn, Seo Young Song, Yo Huh, Hyuk-Jae Chang

**Affiliations:** 1 Division of Trauma Surgery, Department of Surgery, Ajou University School of Medicine, 164 Worldcup-ro, Yeongtong-gu, Suwon, 16499 Korea; 2 Gyeonggi South Regional Trauma Center, Ajou University Hospital, Suwon, Korea; 3 Division of Cardiology, Severance Cardiovascular Hospital, Yonsei University College of Medicine, Yonsei University Health System, Seoul, Korea

**Keywords:** Triage, Emergency medical services,, Injury Severity Score,, Trauma center

## Abstract

**Background:**

We evaluated the accuracy of the prehospital Field Triage Decision Scheme, which has recently been applied in the Korean trauma system, and the factors associated with severe injury and prognosis at a regional trauma center in Korea.

**Methods:**

From 2016 to 2018, prehospital data of injured patients were obtained from the emergency medical services of the national fire agency and matched with trauma outcomes at our institution. Severe injury (Injury Severity Score > 15), overtriage/undertriage rate, positive predictive value, negative predictive value, and accuracy were reviewed according to the triage protocol steps. A multivariate logistic regression analysis was performed to identify influencing factors in the field triage.

**Results:**

Of the 2438 patients reviewed, 853 (35.0%) were severely injured. The protocol accuracy was as follows: step 1, 72.3%; step 2, 65.0%; step 3, 66.2%; step 1 or 2, 70.2%; and step 1, 2, or 3, 66.4%. Odds ratios (OR) (95% confidence interval [CIfor systolic blood pressure < 90 mmHg (3.535 [1.920–6.509]; *p* < 0.001), altered mental status (17.924 [8.980–35.777]; *p* < 0.001), and pedestrian injuries (2.473 [1.339–4.570], *p* = 0.04) were significantly associated with 24-h mortality. Penetrating torso injuries (7.108 [4.108–12.300]; *p* < 0.001); two or more proximal long bone fractures (4.134 [2.316–7.377]); *p* < 0.001); crushed, degloved, and mangled extremities (8.477 [4.068–17.663]; *p* < 0.001); amputation proximal to the wrist or ankle (42.964 [5.764–320.278]; *p* < 0.001); and fall from height (2.141 [1.497–3.062]; *p* < 0.001) were associated with 24-h surgical intervention.

**Conclusion:**

The Korean field triage protocol is not yet accurate, with only some factors reflecting injury severity, making reevaluation necessary.

## Background

Prehospital triage is one of the most important components of trauma systems [1, 2]. Along with the establishment of a trauma system, 119 emergency medical services (EMS) of the national fire agency in Korea have adopted the Field Triage Decision Scheme proposed by the Centers for Disease Control and Prevention (CDC), United States. The Field Triage Decision Scheme consists of four steps with varying criteria: physiological criteria (step 1), anatomical factors (step 2), mechanism of injury (step 3), and factors specific to certain groups (step 4) [3]. According to the CDC guidelines, injured patients who meet the physiological and anatomical criteria should be transported preferentially to a facility “with the highest level of care within a defined trauma system.” However, according to step 3, patients need not be transferred to the highest-level trauma center, depending on the trauma system. Patients who meet the step 4 criteria may be transported to a facility capable of timely, thorough evaluation and initial management of potentially serious injuries [3]. The difference between the CDC guidelines and the Korean guidelines is that patients in steps 1 and 2 are to be transferred to the highest-level trauma center in the CDC guidelines, while the Korean guidelines defined even steps 3 and 4 as severe trauma with the same principle applied.

In 1997, Kim et al. reported that the rate of preventable trauma death in Korea was approximately 50% [4]. This rate has slightly declined but remained at over 30% in 2010. To decrease the rate of preventable trauma death, the government initiated a new Korean trauma system by establishing 17 regional trauma centers, each equivalent to a level-1 trauma center in the US [5]. However, despite the growing number of trauma centers, these efforts by the government have not reached the prehospital system. Unfortunately, the government plan has neither guidelines nor specified designations for sub-level trauma centers. Therefore, patients meeting step 3 criteria are transferred to a regional trauma center.

Recently, some regional trauma centers showed better outcomes among severely injured patients than non-trauma centers [5–7]. Furthermore, patients transported directly to regional trauma centers had better outcomes than those transferred to other facilities [8]. The saying, “the right person, the right place, the right time,” is very important in the triage of trauma patients.

The accuracy of the field triage, especially mechanism of injury (step 3), is controversial. In addition, accuracy may vary according to national and/or regional characteristics; hence, many countries/regions have their own field triage guidelines. Although the accuracy of CDC field triage protocols in selecting severely injured patients has been studied in the US [9], the accuracy of the adopted CDC field triage could be different in Korea. Hence, we investigated the accuracy of the current Field Triage Decision Scheme, as well as the influencing factors, at a single institution with a regional trauma center in Korea. The primary outcome was the accuracy of the adopted CDC Field Triage Decision Scheme at our institution, and the secondary outcome was the identification of factors influencing severe injury and 24-h mortality.

## Methods

Our institution is a tertiary hospital operating a level-1 trauma center and a regional emergency medical center with 100,000 annual patient visits. It is located in the city of Suwon, a metropolitan area, which is approximately 30 km from Seoul and serves a population of 1.3 million. Our trauma center also covers the Southern Gyeonggi Province that has a population of 9 million and registers approximately 2,700 trauma patients per year. We retrospectively reviewed prehospital information and trauma outcomes of patients directly transported by the EMS to our hospital during the period from 2016 to 2018.

Of the total 3150 patients, 712 patients aged < 18 years who were dead on arrival or had missing data (such as air transport or reduplication) were excluded, and the data of the remaining 2438 patients were reviewed.

In accordance with the guidelines, upon encountering patients with various mechanisms of injury, EMS providers at the scene were required to complete “the detailed prehospital trauma sheets for severe injury,” including traffic accidents, falls, penetrating injuries, farm machinery injuries, and other blunt traumas, regardless of injury severity. EMS providers were able to predict and fill this information. Based on the information from the EMS sheet, we matched prehospital information with hospital outcomes. Using these matched data, we evaluated the predictability of severity and early death and identified the influencing factors.

Severe injury was defined as an Injury Severity Score (ISS) of > 15. The accuracy of the field triage scheme was calculated based on its ability to discriminate severe injury as determined by physiological criteria (step 1), anatomical criteria (step 2), and injury mechanisms (step 3). Overtriage rates and undertriage rates were reviewed. Overtriage was defined as satisfying the field triage criteria but with the absence of severe injury. Undertriage was defined as not satisfying the field triage criteria but having severe injury. Out of the 16 subitems of the Field Triage Decision Scheme, 14 subitems, except EMS judgment (of step 4) and respiratory rate (of step 1), which may provide unreliable information, were set up for prehospital information. The primary and secondary outcomes were determined by construction of a fourfold table to calculate accuracy, sensitivity, specificity, negative predictive value (NPV), and positive predictive value (PPV).

Multivariate logistic regression was performed to identify factors associated with prehospital information, injury severity, 24-h mortality, and 24-h surgical intervention. All statistical analyses were performed using IBM SPSS (version 25; IBM Corp., Armonk, NY, USA).

### Ethics statement

This study was approved by the Institutional Review Board (IRB) of Ajou University Hospital (IRB No. AJIRB-MED-MDB-19–374). The need for obtaining informed consent was waived by the board because of the observational nature of the study.

## Results

### General characteristics

In total, 853 (35.0%) patients were considered to have severe injury. Most patients were male (71.9%), and their mean (± standard deviation) age was 50.9 (± 18.0) years. The most common injury mechanism was traffic accidents (53.9%). A total of 1161 patients (47.6%) satisfied the field triage decision criteria (Table 1).

**Table 1:** General characteristics of trauma patients transported by the EMS of the firefighting agency to Ajou Trauma Center from 2016 to 2018

Variable	Value
Sex	
Male, n (%)	1752 (71.9%)
Age, y (mean ± standard deviation)	50.9 ± 18.0
Injury mechanism	
Traffic accident, n (%)	1313 (53.9%)
Fall, n (%)	505 (20.7%)
Slip down, n (%)	271 (11.1%)
Struck, n (%)	99 (4.1%)
Machine, n (%)	73 (3.0%)
Penetrating, n (%)	146 (6.0%)
Other/Unknown, n (%)	31 (1.3%)
Injury Severity Score (mean ± standard deviation)	13.3 ± 11.3
24-h mortality, n	63 (2.6%)
Final mortality, n	167(6.8%)
Triage	
Step 1, n (%)	471 (19.3%)
Step 2, n (%)	296 (10.5%)
Step 3, n (%)	910 (32.3%)
Step 1 or step 2, n (%)	664 (27.2%)
Step 1 or step 2 or step 3, n (%)	1161 (47.6%)

### Accuracy of the Field Triage Decision Scheme

Patients who only satisfied the physiological criteria were accurately triaged 72.3% of the time, while 31.2% and 26.9% were over- and undertriaged, respectively. Sensitivity and specificity were 38.0% and 90.7%, respectively; PPV was 68.9% and NPV was 73.1%.

The accuracy of the group who met the physiologic criteria and/or anatomical criteria was 70.3%, while 40.5% and 25.8% were over- and undertriaged, respectively. Additionally, sensitivity and specificity were 46.3% and 83.0%, respectively; PPV and NPV were 59.5% and 74.2%, respectively.

The accuracy for patients who satisfied any of the physiological criteria and anatomical criteria and dangerous injury mechanism was 66.4%. The overtriage rate, undertriage rate, sensitivity, specificity, PPV, and NPV were 48.6%, 20.0%, 70.0%, 64.4%, 51.4%, and 80.0%, respectively. Interestingly, patients who satisfied the physiological criteria had the highest PPV and accuracy (Table 2).

**Table 2:** Overtriage and undertriage rates, sensitivity, specificity, PPV, NPV, and accuracy of field triage decision factors for each step of the CDC criteria

%	Overtriage	Undertriage	Sensitivity	Specificity	PPV	NPV	Accuracy
STEP 1	31.2	26.9	38.0	90.7	68.8	73.1	72.3
STEP 2	50.2	33.0	16.6	91.0	49.8	67.0	65.0
STEP 3	48.2	26.6	49.5	75.2	51.8	73.4	66.2
STEPS 1 + 2	40.5	25.8	46.3	83.0	59.5	74.2	70.2
STEPS 1 + 2 + 3	48.6	20.0	70.0	64.4	51.4	80.0	66.4

*CDC* Centers for Disease Control and Prevention, *PPV* positive predictive value, *NPV* negative predictive value

### Factors associated with severe injury, 24-h mortality, and 24-h emergency surgical intervention for field triage decision

Of the 14 subitems, multivariate analysis revealed that penetrating injuries (odds ratio (OR) 95% confidence interval [CI], 0.791 [0.451–1.390]; *p* = 0.416), mangled extremities (OR [95% CI], 0.560 [0.276–1.138]; *p* = 0.109), and open skull fractures (OR [95% CI], 1.450 [0.540–3.897]; *p* = 0.461) were not associated with severe injury (Table 3).

**Table 3:** Factors associated with severe injury (ISS > 15) for field triage decision

	ISS > 15		
	OR	95% CI	*p*
Altered mental status	**5.135**	**3.974–6.635**	** < 0.001**
SBP < 90 mmHg	**2.107**	**1.380–3.218**	**0.001**
Penetrating torso injuries	0.791	0.451–1.390	0.416
Chest wall deformity	**20.116**	**2.464–164.208**	**0.005**
Two or more proximal long bone fractures	**2.419**	**1.277–4.582**	**0.007**
Crushed, degloved, mangled extremities	0.560	0.276–1.138	0.109
Amputation of proximal to wrist or ankle	**3.823**	**1.544–9.466**	**0.004**
Pelvic bone fracture	**2.780**	**1.208–5.943**	**0.015**
Open, depressed skull fractures	1.450	0.540–3.897	0.461
Paralysis	**5.463**	**2.372–12.584**	** < 0.001**
Fall from height	**5.280**	**3.493–7.981**	** < 0.001**
High-risk auto crush	**2.009**	**1.526–2.644**	** < 0.001**
Auto vs. bicycle/pedestrian crush	**1.689**	**1.246–2.289**	**0.001**
High-speed auto bicycle crush	**2.002**	**1.390–2.884**	** < 0.001**

*OR* odds ratio, *CI* confidence interval, *ISS* Injury Severity Score, *SBP* systolic blood pressure

Systolic blood pressure of < 90 mmHg (OR [95% CI], 3.535 [1.920–6.509]; *p* < 0.001), altered mental status (OR [95% CI], 17.924 [8.980–35.777]; *p* < 0.001), and pedestrian accidents (OR [95% CI], 2.473 [1.339–4.570]; *p* = 0.04) were found to be significantly associated with 24-h mortality (Table 4). Penetrating torso injuries (OR [95% CI], 7.108 [4.108–12.300]; *p* < 0.001); two or more proximal long bone fractures (OR [95% CI], 4.134 [2.316–7.377]; *p* < 0.001); crushed, degloved, and mangled extremities (OR [95% CI], 8.477 [4.068–17.663]; *p* < 0.001); amputations proximal to the wrist or ankle (OR [95% CI], 42.964 [5.764–320.278]; *p* < 0.001); and falls from height (OR [95% CI], 2.141 [1.497–3.062]; *p* < 0.001) were associated with 24-h surgical interventions (Table 5).

**Table 4:** Factors associated with 24-h mortality in field triage decision

Field Triage Decision factors	OR	95% CI	*p*
Altered mental status	15.919	7.857–32.253	< 0.001
SBP < 90 mmHg	3.614	1.928–6.777	< 0.001
Auto vs. bicycle/pedestrian crush	3.327	1.667–6.641	0.001

95% CI, 95% confidence interval; *OR* odds ratio, *SBP* systolic blood pressure

**Table 5:** Factors associated with 24-h surgical interventions in field triage decision

Field Triage Decision factors	OR	95% CI	*p*
Penetrating torso injuries	7.108	4.108–12.300	< 0.001
Two or more proximal long bone fractures	4.134	2.316–7.377	< 0.001
Crushed, degloved, mangled extremities	8.477	4.068–17.663	< 0.001
Amputation of proximal to the wrist or ankle	42.964	5.764–320.278	< 0.001
Fall from height	2.141	1.497–3.062	< 0.001

95% CI, 95% confidence interval; *OR* odds ratio

## Discussion

In this study, we found that the accuracy of the adopted CDC field triage protocol had a high undertriage rate at our institution and that some subitems were not associated with severe injury. Care at trauma centers can increase the survival rate of trauma patients. However, because resources at trauma centers are limited, admission of patients to such centers should be decided carefully. To achieve efficiency, it is necessary to manage the number of overtriaged and undertriaged patients. The American College of Surgeon-Committee of Trauma (ACS-COT) recommends an undertriage rate below 5% and overtriage rate of 25–30% [10].

Unfortunately, similar to the situation at our institution, other institutions also face difficulties in following the ACS-COT overtriage/undertriage recommendation. In the Netherlands, Laarhoven et al. reported overtriage and undertriage rates of 39.5% (95% CI, 36.9–42.1) and 10.9% (95% CI, 7.4–15.7), respectively [11]. In a systematic review of 21 studies, the undertriage rates ranged from 1% to 71.9% and overtriage rates ranged from 19 to 79% [12]. Nevertheless, efforts to reduce the undertriage/overtriage rate by adjusting the field triage variables are important. Newgard et al. reported that the overall sensitivity and specificity of the CDC field triage, including in children with an ISS of ≥ 16, were 85.8% (95% CI, 85.0–86.6) and 68.7% (95% CI, 68.4–68.9), respectively [13]. Another study by Newgard et al. revealed that the sensitivity and specificity of the CDC field triage protocol were 66.2% (95% CI, 60.2–71.7) and 87.8% (95% CI, 87.7–88.0), respectively [14]. This prospective, multicenter study argues that the CDC field triage guideline is relatively insensitive in identifying severely injured patients and the elderly, thereby warranting improvement.

Nevertheless, not all countries and regions follow these guidelines. Each region and environment have different guidelines and have undergone evaluation of their effectiveness. Cassignol et al. evaluated a correlation between the French field triage criteria and the ISS at a trauma center in France [15]. Nowakowski et al. compared the Polish qualification criteria for trauma centers to the CDC guidelines and argued that there was no difficulty in predicting outcomes in patients with an ISS of ≥ 15 despite the mechanism of injury not being considered in the Polish criteria [16]. Stephen et al. introduced the Circulation, Respiration, Abdomen, Motor, Speech (CRAMS) scale and emphasized the need for a simpler scale with easier application at the scene [17]. Moreover, Clemmer et al. reported the CRAMS scale as an easy scoring system for physiological criteria and torso injury, showing the usefulness of this tool in deciding whether to transport a patient to a level-1 trauma center or not [18].

Numerous studies on prehospital triage criteria have been conducted abroad, but only a few such studies have been conducted in Korea. In the present study, we studied the accuracy of the adopted CDC Field Triage Decision Scheme by matching prehospital information with hospital outcomes of patients who visited our institution. Most of the items, including physiological criteria, anatomical findings, and mechanisms of injury, were related to ISS of ≥ 15, but some items were not. In particular, the accuracy and PPV of step 3 were only 66.2% and 51.8%, respectively; therefore, some danger mechanisms need to be considered in the field triage guidelines when transferring patients to the highest-level trauma center. Points related to this issue require detailed discussion and consultation, and may have to change depending on different criteria and cases.

Another important issue is that it is difficult to base the definition of severe trauma on an ISS of > 15, and it is not easy to identify injury severity at the scene. There is a clear difference in assessing the severity between prehospital information and ISS because ISS score is determined based on anatomical, surgical, and autopsy findings after treatment at hospitals [19–22]. Because severe trauma was defined as an ISS of > 15 in this study, it was thought that this score did not reflect all situations in the field. To compensate for this, Champion et al. devised the Trauma Score using physiological data in 1981 and then revised it to create the Revised Trauma Score in 1989 [23, 24]. However, the direct application of this scoring system in the field is complicated [21]. Therefore, what factors to consider when judging the injury severity of trauma patients in the field are of inevitable concern for the emergency department and EMS providers.

This study has several limitations. First, triaging is a regional problem and not restricted to a single hospital. In fact, every year, more than 100,000 “detailed prehospital trauma sheets for severe injury” were documented in the Gyeonggi Province, but only a few were included in this study. Second, our institution has a level-1 trauma center; therefore, it has a higher proportion of severe trauma patients than that seen in other hospitals, which might have resulted in selection bias. Third, we defined severe injury as an ISS of > 15, thereby excluding patients with an ISS of < 15 who needed acute care. Many studies defined severe trauma as an ISS of > 15 [25–27], but even if the ISS is not high, serious trauma requiring emergency surgery or intervention cannot be completely excluded. Jacob et al. reported that the need for trauma intervention was a better definition for major trauma than ISS [28]. Therefore, it is necessary to adjust the future protocols accordingly. Although more than half of the included patients did not satisfy the field triage decision criteria, they were admitted to a level-1 trauma center for inpatient treatment. This implies possible omissions from the records of the EMS provider, which may indicate that the triage protocol itself does not fully reflect the severity of patient injuries. Education and quality control for the provided checkbox are required, and further prospective studies may be necessary. As previously mentioned, in an effort to reduce preventable trauma deaths, the Korean government has established 17 regional trauma centers. However, field triage and prehospital care have remained inadequate and were, therefore, identified as problems in the Korean trauma system. The Korean trauma system needs a triage tool suitable for the current situation in the country, and low-level trauma centers need to improve the overtriage/undertriage ratio and reduce the rate of preventable trauma deaths.

Nevertheless, this study is the first to investigate the accuracy and problems of prehospital field triage in Korea by matching prehospital and hospital information. Our findings suggest that initial consciousness and vital signs are highly correlated with early death. Several anatomical factors were helpful in predicting the need for surgery within 24 h. However, determining which injury mechanisms are negligible is difficult, leading to confusion. Based on these findings, the field triage protocol should be updated regularly and evaluated for regional characteristics. For this, it is essential to establish a trauma outcome database (such as the Korean Trauma Data Bank) that includes data from more facilities, such as non-trauma centers. Multiple tasks ranging from field triage to surgical treatment, intensive care, geriatric trauma, rehabilitation, and return to society need to be focused on by trauma centers to promote the regional trauma system. Among them, field triage and appropriate transport can make substantial contributions in reducing preventable trauma deaths.

## Conclusion

Prehospital information and outcomes from various facilities should be analyzed to ensure the application of an appropriate Field Triage Decision Scheme for each regional trauma system.

## Data Availability

Among the data sets analyzed during the current study, prehospital data were provided by the Korean Fire agency. Data used and/or analysed during the current study available from the corresponding author on reasonable request.

## Abbreviations

EMS: Emergency medical services
CDC: Centers for Disease Control and Prevention
ISS: Injury Severity Scale
CRAMS: Circulation, respiration, abdomen, motor, speech
PPV: Positive predictive value
NPV: Negative predictive value
OR: Odds ratio
CI: Confidence interval
ACS-COT: American College of Surgeon-Committee of Trauma
